# Case Report: Acute polymorphic psychosis and NMDA-R IgG antibodies in serum: a follow-up case study

**DOI:** 10.3389/fimmu.2025.1630357

**Published:** 2025-08-25

**Authors:** Katharina von Zedtwitz, Judith Weiser, Raphael J. Dressle, Simon J. Maier, Bernd Feige, Kathrin Nickel, Nils Venhoff, Katharina Domschke, Joachim Brumberg, Sebastian Rauer, Ludger Tebartz van Elst, Luciana Hannibal, Harald Prüss, Alexander Rau, Dominique Endres

**Affiliations:** ^1^ Department of Psychiatry and Psychotherapy, Medical Center - University of Freiburg, Faculty of Medicine, University of Freiburg, Freiburg, Germany; ^2^ Department of Rheumatology and Clinical Immunology, Medical Center - University of Freiburg, Faculty of Medicine, University of Freiburg, Freiburg, Germany; ^3^ German Center for Mental Health (DZPG), Partner Site Berlin, Berlin/Potsdam, Germany; ^4^ Department of Nuclear Medicine, Medical Center - University of Freiburg, Faculty of Medicine, University of Freiburg, Freiburg, Germany; ^5^ Department of Neurology, Medical Center - University of Freiburg, Faculty of Medicine, University of Freiburg, Freiburg, Germany; ^6^ Laboratory of Clinical Biochemistry and Metabolism, Department of General Pediatrics, Adolescent Medicine and Neonatology, Medical Center - University of Freiburg, Faculty of Medicine, University of Freiburg, Freiburg, Germany; ^7^ Department of Neurology and Experimental Neurology, Charité - Universitätsmedizin Berlin, Berlin, Germany; ^8^ German Center for Neurodegenerative Diseases (DZNE) Berlin/Potsdam, Berlin, Germany; ^9^ Department of Neuroradiology, Medical Center - University of Freiburg, Faculty of Medicine, University of Freiburg, Freiburg, Germany

**Keywords:** autoimmune, inflammation, brain, immunotherapy, autoimmune encephalitis

## Abstract

**Introduction:**

Anti-N-methyl-D-aspartate receptor (NMDA-R) encephalitis is a neuropsychiatric disorder with additional psychiatric features caused by NMDA-R immunoglobulin G (IgG) antibodies in cerebrospinal fluid (CSF). This report presents the follow-up of a patient in whom we assumed mild NMDA-R encephalitis in the first psychotic episode.

**Case study:**

A patient with a prior episode of an acute polymorphic psychotic syndrome relapsed five and a half years later following a severe COVID-19 infection. Serum NMDA-R antibodies were again detected with a titer of max. 1:320 using fixed-cell-based assays, but conventional magnetic resonance imaging (MRI), electroencephalography (EEG), and CSF findings were largely normal. NMDA-R antibody levels in serum decreased to 1:80 after approximately one month without immunotherapy. [^18^F]fluorodeoxyglucose positron emission tomography (FDG-PET) still revealed pronounced metabolism of the association cortices (clearly more pronounced in the first episode with an encephalitis-like pattern at that time). Advanced MRI analyses including diffusion microstructure imaging (DMI) showed frontal and thalamic microstructural alterations compatible with edematization (but also far less accentuated than in the first episode). Further advanced antibody tests of CSF (approx. 1 month after symptom onset) using a live-cell-based and different tissue-based assays were negative for NMDA-R IgG antibodies. Research mass spectrometry of the CSF identified neurotransmitter-precursor shortages, increased turnover of tryptophan into quinolinic acid, and low-glucose/lactate levels. Immunotherapy (performed after the initial assumption of an autoimmune cause) with steroids led to clinical improvement of residual symptoms. After approximately three months, NMDA-R IgG serum antibodies were no longer detectable; however, FDG-PET/DMI follow-up revealed no relevant changes.

**Discussion:**

The international consensus criteria for a probable/definite diagnosis of NMDA-R encephalitis or autoimmune psychosis were not fulfilled, especially as no NMDA-R IgG antibodies were identified in CSF using different antibody assays and EEG/CSF routine findings were inconspicuous. NMDA-R encephalitis was therefore not diagnosed (as initially suspected). Independent of the NMDA-R IgG antibodies, there were possible signs of an autoimmune process. For a better understanding of similar patients, multimodal diagnostic approaches including complementary antibody tests could be promising.

## Introduction

Anti-N-methyl-D-aspartate receptor (NMDA-R) encephalitis is characterized by a variety of symptoms, including psychiatric symptoms, cognitive dysfunction, speech dysfunction, seizures, movement disorder, decreased levels of consciousness, autonomic dysfunction, and central hypoventilation (1, 2). The key diagnostic finding is the detection of anti-GluN1 immunoglobulin G (IgG) NMDA-R antibodies in cerebrospinal fluid (CSF) in combination with other abnormalities in routine diagnostics (e.g., with electroencephalography [EEG] pathologies in approx. 90% and CSF changes in approx. 80%) (1–3). Predominant or isolated psychiatric manifestations, or so-called autoimmune psychosis cases, have also recently been described (4). NMDA-R IgG antibodies were also positive in the serum of 0.59% of patients with schizophreniform psychosis and in 0.62% of healthy controls using fixed-cell-based assays (5). A recent study comparing patients with and without NMDA-R antibodies in serum found that the antibody-positive patients had less pronounced negative symptoms and relatively good psychosocial functioning (6). Commercially available fixed-cell-based assays are mostly used for antibody testing (1, 2). In addition, cell-based assays on living cells (live-cell-based assays) and tissue-based assays on brain slices are available in research laboratories (2, 7). In 2019, we reported on a patient with acute polymorphic psychosis, serum NMDA-R IgG antibodies in fixed-cell-based assays (with unremarkable CSF findings), and encephalitic FDG-PET findings; at that time, we suspected mild NMDA-R encephalitis (8). This paper provides a critical follow-up on that patient.

## Case study

**Figure 1:**
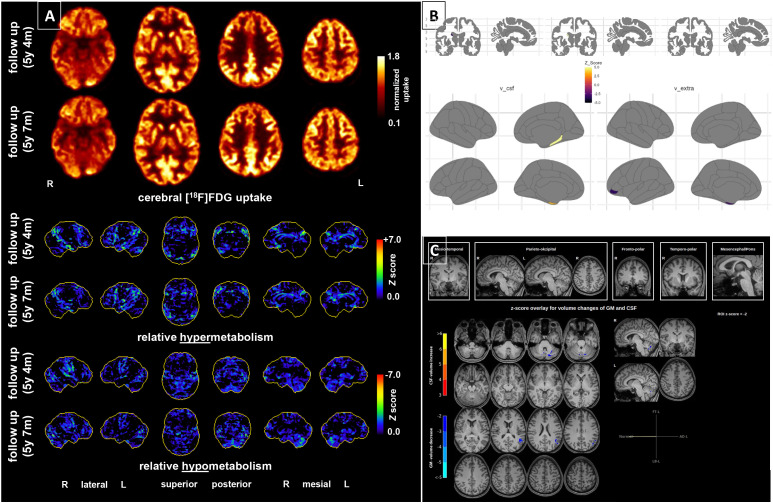
Brain imaging findings during the relapse five and a half years after the first psychotic episode. **(A)** [^18^F]Fluorodeoxyglucose (FDG) positron emission tomography (PET) in the second episode (before steroids) revealed low to moderate hypermetabolism of the association cortices and cerebellum. The follow-up did not show relevant improvement in the findings. R=right, L=left. **(B)** Advanced magnetic resonance imaging (MRI) with diffusion microstructure imaging (DMI) in the second episode (before steroids) showed frontal and thalamic microstructural alterations of grey matter integrity (comparison of the studied patient with 24 sex- and age-matched healthy controls, mean age 27.38 ± 4.48 years. Data were parcellated according to the ASEG atlas (top row) and the Desikan-Killiany (bottom row). The cerebral blood flow using arterial spin labeling MRI was normal (not shown). v=volume. **(C)** MRI showed no macrostructural volume loss (https://www.veobrain.com/?page=veomorph) in the second episode.

**Figure 2:**
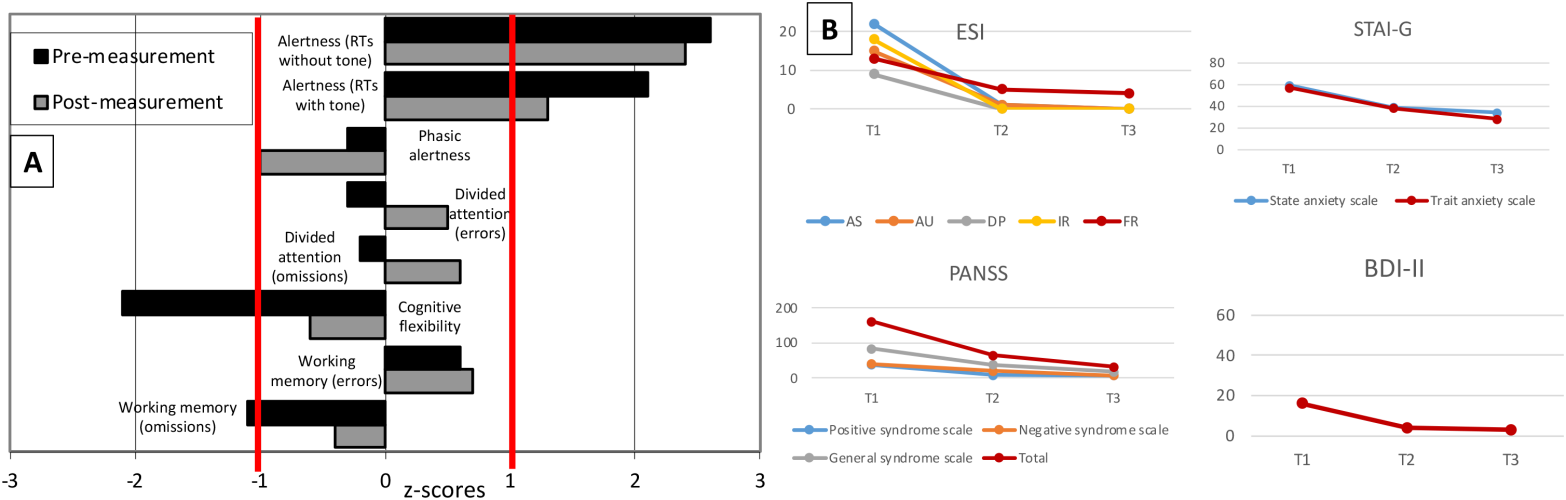
Neuropsychological and psychometric tests during the relapse five and a half years after the first psychotic episode. **(A)** Neuropsychological test results before and after steroid treatment (23 days after starting steroids) show normalized results in terms of mental flexibility and working memory. There were no residual impairments in any of the functions tested at the post-treatment measurement. Z-scores are shown (reference: -1 to +1). RTs=reaction times. **(B)** Structured questionnaires (ESI, Eppendorf Schizophrenia Inventory; PANSS, Positive and Negative Syndrome Scale; STAI-G, State-Trait Anxiety Inventory; BDI-II, Beck Depression Inventory) were used for psychometric follow-up. As the patient had already been referred in the course of clinical improvement, symptoms were assessed retrospectively at the beginning of the acute episode (T1), then before (T2) and after (T3) steroid pulse treatment (20 days after starting steroids).

Approximately 10 days after her third COVID-19 infection, the 28-year-old female patient, who gave her written informed consent for this follow-up report, had an acute polymorphic psychotic relapse with delusional symptoms, confused behavior, affective instability, sensory overload, and cognitive symptoms with initial circulatory problems, tiredness, and headache. She had previously been vaccinated against COVID-19 three times with an mRNA vaccine. A detailed description of her medical history from the patient’s perspective is provided in **Box 1**. **Figures 1**, **2** and **Table 1** summarize the diagnostic findings.

**Table 1:** Diagnostic findings (second episode).

Serum antibodies, immunological markers and serologies	
Anti-thyroid antibodies (against TPO, TG and TSH-receptor)	Normal
ANAs (*on HEp-2 cells*), ANCAs (*on EthOH-/formalin-fixed neutrophils*), APAs Rheumatoid factor/anti-CCP	Normal
Complement factors (C3, C4)	Normal
IgG, IgM, and IgA levels	Normal
CRP	<0.6 mg/l (ref.: <5 mg/l)
Serology for Lyme disease or lues	Negative
Serologies (CMV, EBV, HAV, HBV, HCV, HIV, tuberculosis)	CMV and EBV seropositivity, anti-Hbs increased in the post-vaccination state
GAD65 antibodies using CLIA	Negative
Well-characterized neuronal IgG antibodies against intracellular antigens using an immunoblot	Negative
Well-characterized neuronal IgG cell surface antibodies in indirect immunofluorescence test using fixed-cell-based assays	NMDA-R antibodies positive (max. titer max. 1:320)
Anti-NMDA-R IgG antibodies in indirect immunofluorescence using live cell-based assays and tissue-based assay (reference laboratory, Prof. Dalmau, Barcelona, Spain)	Negative
Anti-MOG/AQP4-IgG antibodies	Negative
Tissue-based assay on unfixed murine brain slices (Prof. Prüss; Charité Berlin, Germany)	Negative
Cerebrospinal fluid	
White blood cell count	1/µL (ref.: <5/µL)
Protein concentration	185 mg/L (ref.: <450 mg/L)
Albumin quotient	2.6 (ref.: <7.7)
IgG-index	0.48 (ref.: <0.7)
Oligoclonal bands in serum/CSF	Negative/negative
Local IgG/IgA/IgM synthesis	None (ref.: <10%)
Well-characterized neuronal IgG cell surface antibodies in indirect immunofluorescence test using fixed-cell-based assays	Negative
Anti-NMDA-R IgG antibodies in indirect immunofluorescence using live cell-based assays and tissue-based assay (reference laboratory, Prof. Dalmau, Barcelona, Spain)	Negative, atypical pattern from CSF material in the live cell-based assay
Tissue-based assay on unfixed murine brain slices (Prof. Prüss; Charité Berlin, Germany)	Negative
Neurometabolic measurements (without medication; PD Dr. Hannibal, Core Facility Metabolomics, Medical Center - University of Freiburg, Germany)	The profiling of metabolites in CSF revealed low levels of glutamate (0.56 ± 0.11 µM; normal range: 33 ± 7 µM), glutamine (70 ± 14 µM; normal range: 440 ± 80 µM), serotonin (0.0034 ± 0.0007 µM; normal range: 0.82 ± 0.48 µM), tyrosine (0.90 ± 0.17 µM; normal range: 10 ± 4 µM), urea (1090 ± 218 µM; normal range: 3000-6500 µM), glucose (1790 ± 358 µM; normal range: 2700-4400 µM), lactate (756 ± 151 µM; normal range: 1590 ± 330 µM) and kynurenine (0.017 ± 0.003 µM; normal range: 0.1-0.5 µM), low-normal GABA (0.098 ± 0.020 µM; normal range: 0.127 ± 0.0052 µM), and markedly elevated tryptophan (9.2 ± 1.8 µM; normal range: 2 ± 1 µM) and quinolinic acid (1.04 ± 0.21 µM; normal range: <0.050 µM).
MRI of the neurocranium	
Visual inspection	Largely normal
Automated morphometry	Normal, no volume loss
Cerebral blood flow using arterial spin labeling	Normal
Diffusion microstructure imaging (DMI)	Alterations of gray matter integrity in frontal and thalamic areas compatible with edematization upon comparison with an age- and sex-matched cohort of healthy controls (n=24 female with mean age of 27.4 ± 4.5 years). This pattern resembles the one observed in 2019 (though only analyzed retrospectively), however with less pronounced alterations. The follow-up after approx. three months showed slightly increase in frontal edematization.
EEG	
Visual analyses	Normal
Independent component analysis	Initially normal - minimal increase in IRDA/IRTA rates directly after steroid treatment
FDG-PET	
Brain	***Initially:*** Pronounced metabolism of the association cortices. The voxel-based statistical analysis showed a hypermetabolism of the association cortices with the following Z-scores: frontal Z-score +2 to +3, parietooccipital Z-score around +4, cerebellar Z-score up to +2.
Whole-body	***Approx. three months follow-up:*** The visual findings show a largely constant cortical radioglucose storage with emphasis on the association cortices compared to the initial examination. In addition, largely regular radioglucose storage in the subcortical and cerebellar areas were identified. In the additional voxel-based statistical evaluation to the preliminary examination in the second episode, largely constant, at most slightly decreasing hypermetabolism parietooccipitally (Z-score around +3). No evidence of a malignant tumor.
Ophthalmological tests	
Optical coherence tomography	Normal
Electroretinography	Normal

Well-characterized neuronal IgG antibodies against intracellular antigens were analyzed using an immunoblot (c.f. 9; Ravo PNS 11 Line Assay^®^, Freiburg, Germany). Well-characterized neuronal IgG cell surface antibodies (including NMDA-R IgG antibodies) were tested using indirect immunofluorescence on commercially available, fixed, cell-based assays (c.f. 9; “Mosaic 6” from Euroimmun^®^, Lübeck, Germany). In addition, a live-cell-based assay for anti-NMDA-R IgG antibodies using indirect immunofluorescence (10) and a tissue-based assay screening on rat brain slices (10) were performed in the reference laboratory (Prof. Dalmau, Barcelona, Spain). Finally, a tissue-based assay on unfixed murine brain slices was added (11, 12; Autoimmune Encephalopathies Laboratory, German Center for Neurodegenerative Diseases and Charité Berlin; Prof. Prüss, Berlin, Germany). ADHD, attention-deficit/hyperactivity disorder; ANAs, antinuclear antibodies; ANCAs, anti-neutrophil cytoplasmic antibodies; APAs, antiphospholipid antibodies; AQP4, aquaporin-4; BDI-II, Beck Depression Inventory II; CAARS, Conners’ Adult ADHD Rating Scales; CLIA, chemiluminescence immunoassay; CCP, cyclic citrullinated peptide; CMV, cytomegalovirus; CRP, C-reactive protein; CSF, cerebrospinal fluid; CT, computed tomography; DMI, diffusion microstructure imaging; DNaseB, deoxyribonuclease B; DSM-IV, Diagnostic and Statistical Manual of Mental Disorders; DTI, diffusion tensor imaging; EBV, Epstein-Barr virus; ECG, electrocardiography; EEG, electroencephalography; FDG-PET, [^18^F]fluorodeoxyglucose-positron emission tomography; GAD65, glutamic acid decarboxylase 65-kilodalton isoform; HAV, hepatitis A virus; HBV, hepatitis B virus; HCV, hepatitis C virus; HIV, human immunodeficiency virus; IgA/G/M, immunoglobulin A/G/M; IRDA, intermittent rhythmic delta activity; MOG, myelin oligodendrocyte glycoprotein; MRI, magnetic resonance imaging; MRZ, antibody indices against measles, rubella, and varicella zoster virus; PCR, polymerase chain reaction; ref., reference; SD, standard deviation; TB, tuberculosis; TG, thyroglobulin; TPO, thyroid peroxidase; TSH, thyroid-stimulating hormone; V, volume; VZV, varicella zoster virus.

Box 1 **Medical history from the patient´s perspective.**

  ➢ **July 8, 2024:** Onset of COVID-19 infection with positive test. Symptoms: High fever up to 40°C, headache, cough, aching limbs, cold symptoms, feeling of weakness, sore throat, thick lymph nodes on the neck.

➢ **July 16, 2024:** Negative corona test, only mild cold symptoms, on the road to recovery, only a little weak.

➢ **July 17-19, 2024:** Still very slight cold, but otherwise normal life, a little tired.

➢ **July 20, 2024:** Aggravation! Heatwave in Germany (well over 30°C). Feeling of weakness, circulatory instability (sweaty, hot cold hot cold, pale, have to lie down or sit down all the time, later headache, restless, stressed, panic a little, but can calm down again).

➢ **July 21, 2024:** It’s getting blurry, it takes me a long time to make an avocado sandwich, I can’t really get out of bed, I forget what I wanted to do. My head rattles without a break, I question everything. At night, the delusions start, my head rattles. Increasing drowsiness, confusion, insomnia. To distract myself, I listen to podcasts, watch the news (which is also about fake news) and unfortunately reality then blurs with my delusions, so that I suddenly think that I’m living in a fake world (suddenly think that my roommate isn’t even a doctor, that I’m not worth living).

➢ **July 22, 2024:** I am confused, call my parents that I am confused, they call an ambulance with the help of a friend (as they are both abroad). I must have called a lot of friends and was very confused. Ambulance arrives, I didn’t speak at all, was apathetic, didn’t move or react (lying in bed with headphones on). But I understood what I was told. But I only reacted when a very familiar voice on the phone told me to do what the emergency doctor said. That’s how I got to the emergency room. From now on I don’t remember everything properly, I have partial blackouts. My blood was taken and a lumbar puncture was performed (was I given propofol)?. I don’t remember that though. I then got super silly, said crazy things, just nonsense. Then, for the rest of the day, I fluctuated between panic, delusions, word-finding problems and completely misconnected things. To the point of pure despair, where I screamed and cried and repeated the same things over and over again for hours. I can’t remember the MRI, but I can remember standing in front of it. I was ranting there, being rude (MRI is supposed to be quicker). After that I calmed down and got a room in the neurology department. I think I’m super intelligent and invented AI and Chatgpt, which was also somehow related to Corona in my mind. I thought I was corona patient no. 1 who infected everyone at carnival and spread it all over the world. I have to find the cure to stop the whole thing, otherwise the whole world will die. I had also found a solution (don’t quite remember the thought process). My solution was somehow that I have to go through pain so that no one else has to die. This is why I pulled my accesses. I just linked a lot of things completely wrong. I also thought a friend of mine gave me a code to decrypt, it was set up something like a medical test and I tried to decrypt it and then I would get in to medical school. Everything suddenly felt like a test. At times I mistook the hospital for a testing station, but at other times I understood that I was in hospital.

➢ **July 23, 2024:** Becoming clearer, trying to understand why I’m here. On the one hand I understand that I am the patient, on the other hand I think this is a test for medical school. I’m misinterpreting everything. I think I have tasks to do. My room neighbor is an old bedridden lady with lots of tubes. She keeps mumbling that she would like to be relieved of her gloves. I try to calm her down and talk to her. I inspect her infusions and try to understand what task I have to do here, but I also realize that something is wrong and that it doesn’t make sense. My father later tells me that I was convinced that my grandmother was lying there and that I had to save her or she would die (which is why I still want to go back to the old double room later). I don’t want to leave the double room because I don’t think I’ll have completed the task for medical school. I want to take the test again, which of course isn’t possible and is nonsense. I have to vomit a few times and want to freshen up, which is interpreted as entrenching myself in the room. In between, I confide in the doctors and then again I don’t. I try to understand everything, but my mind is so strained that I can’t really take it all in and everything happens too quickly. Somehow the doctors convince me to go to a new single room. A lot of my thoughts are about the Middle East conflict at this time. I am convinced that I have to show the world that it is not okay how Jews are treated and I provoke the staff to make a statement. This leads to a fixation, which I fight against. A day later, I told my mother that I didn’t think the restraint was so bad. I was also convinced that I was partly to blame for the Middle East conflict and that I could somehow solve it. My buddy and my father arrive at the hospital around 10 pm. Now I understand all the more that something is really wrong. I’m still half in my warning mood but clearer. They are able to calm me down and convince me to take a tablet, probably lorazepam. My heart is racing, I feel totally dehydrated (I’ve probably hardly eaten anything).

➢ **July 24, 2024:** My parents come to the hospital in the morning. I’m given another prescription and I’m getting clearer and clearer. The judge comes by and I’m still talking a bit confused, but I realize more and more that this is all nonsense and that I’m becoming clearer and clearer. The judge decides that I can be released from the restraint, but that I have to go to a psychiatric ward for a week. The restraints finally come off and I’m really looking forward to being with my family and having a shower, but the ambulance is already waiting for me to take me to the psychiatric ward. I realize that I’ve been totally out of line, that everything I thought was nonsense, and I apologize to the staff, who are very confused and surprised that I’ve suddenly become “normal” so quickly. But then I’m taken to the closed ward. Haven’t had time to talk to my family and loved ones in peace now that I’ve been pretty clear. I’m totally exhausted and broken and have massive headaches and back pain and can only stand it in certain positions. It’s all too much for me, I just want to sleep and go home and be with my loved ones, but I can’t because the ward is closed. They ask me to take risperidone, I don’t want to, but in the end I give in (I felt under a lot of pressure, the senior doctor left no room for discussion). The closed ward is very stuffy, bad smell everywhere, hustle and bustle, someone is constantly disturbing me, doesn’t want to be there. Can only get out with visitors at the beginning. I want to recover, but it’s hard to do that in a triple room with hardly any air and a loud noise level.

➢ I’ll have a lot of visitors **over the next few days** and I’ll feel fine, but otherwise I find it really awful. I tell the doctors to please contact Freiburg. But that will take some time again. Risperidone makes me totally numb. I can’t let my feelings out, I can’t cry. My reaction time is slower and I’m wobbly on my feet, it totally restricts me and doesn’t bring me any benefit.

About five and a half years earlier, the patient had experienced a first acute polymorphic psychotic episode (8). At that time, anti-NMDA-R IgG antibodies with a maximum titer of 1:320 were detected in fixed-cell-based assays; however, her CSF findings were completely normal, and no clear pathological findings were detected by EEG or conventional magnetic resonance imaging (MRI). An abnormal [^18^F]fluorodeoxyglucose positron emission tomography (FDG-PET) scan indicated relative hypermetabolism of the association cortices and relative hypometabolism of the primary cortices; hence, steroid pulse treatment was initiated. That treatment significantly improved (but did not completely normalize) the FDG-PET findings 20 days after initiation of the steroid pulse and led to rapid clinical improvement and full remission in the following maximal six months (8). Over the subsequent five and a half years, the patient remained asymptomatic and successfully completed her university degree.

During the current second episode, the diagnostic workup revealed inconclusive results on conventional MRI and EEG. NMDA-R IgG antibodies were again positive in the serum, with a similar maximum titer of 1:320 (approximately two days after the onset of psychotic symptoms) using a fixed cell-based assay (as was the case in the first episode). The CSF findings were completely normal, and the CSF NMDA-R IgG antibodies, measured with a fixed-cell-based assay, also remained negative. The hypothesis of an autoimmune cause was therefore rejected at another hospital, and the patient was transferred to a psychiatric care hospital.

After transfer to our hospital at a later stage, a comparable constellation of findings was present as in the first episode. FDG-PET again showed alterations (approximately 25 days after symptom onset), with pronounced metabolism of the association cortices. The parietooccipital findings were largely identical to those of the first follow-up 20 days after the beginning of the 2019 steroid pulse treatment but did not reach the overall severity of the very first FDG-PET findings from 2019 (before the first steroid pulse). A decrease in NMDA-R antibody levels to 1:80 (initial titer: 1:320) was detected in serum using a fixed cell-based assay after 30 days without immunotherapy (the CSF analysis during that time again revealed a negative NMDA-R antibody finding). Therefore, we added tissue-based assays on unfixed mouse brain slices (11–13) which revealed unsuspicious findings. A negative tissue-based assay for reactivity against the NMDA-R was also found in the reference laboratory of Prof. Dalmau in Barcelona (2). In addition, the live-cell-based assay in the reference laboratory detected no typical reactivity for NMDA-R IgG antibodies in serum and CSF. Further advanced MRI analyses of microstructural MRI metrics (14, 15) revealed alterations of gray matter integrity in the frontal and thalamic areas compatible with edematization upon comparison with an age- and sex-matched cohort of healthy controls (n = 24 female with mean age of 27.4 ± 4.5 years) (**Figure 1**). This pattern resembles the one observed in 2019 (though only analyzed retrospectively) but was far less accentuated than in the first episode. Cerebral perfusion obtained via arterial spin labeling was normal, and we did not note macrostructural atrophy using automated morphometry (https://www.veobrain.com/?page=veomorph). After routine diagnostic workup, the CSF was subjected to quantitative mass spectrometry analysis of neurometabolites on a research basis. Metabolite extraction, quantification, and quality control were performed as described previously (16–23). The metabolite profile revealed low levels of glutamate, glutamine, serotonin, tyrosine, urea, glucose, lactate and kynurenine, low–normal GABA, and markedly elevated tryptophan and quinolinic acid.

In the second episode, psychotic symptoms already improved after 2–3 days, even without antipsychotics or immunotherapy (however, the patient was receiving benzodiazepines). In the meantime, low-dose risperidone at a maximum of 2 mg per day was prescribed. Positive effects could not be clearly observed. Risperidone was poorly tolerated, with the patient experiencing a feeling of indifference, cognitive deficits, blurry vision (after a few days), and hyperprolactinemia with lactation (after 2–3 weeks). Therefore, risperidone was discontinued. After approximately 30 days, the patient was unmedicated and still affectively unstable, ambivalent, and interpersonally dependent, as well as demonstrating cognitive deficits. Compared to the initial (retrospectively collected) psychopathological scores, there was already a clear improvement. Neuropsychologically, she showed still reduced mental flexibility and below average findings in working memory.

Approximately one month after the current onset of psychotic symptoms, steroid pulse treatment was administered with 5 × 500 mg methylprednisolone. This treatment was offered under the initial hypothesis of an autoimmune cause, based on slightly altered FDG-PET findings, positive serum NMDA-R IgG antibodies in the fixed-cell-based assay, and the patient’s history. The steroids were then tapered off orally for about six weeks. In addition, the patient received cognitive behavioral psychotherapy sessions with a clinical psychologist.

The patient was discharged from the inpatient setting 24 days after initiating steroid treatment. Clinically, there was almost complete remission. Approximately 20 days after the start of steroids, the final psychometric examination was carried out, which revealed normal findings. After 23 days, the neuropsychological follow-up examination also showed completely normal results (**Figure 2**). The serum anti-NMDA-R antibody levels using fixed-cell-based assays still resulted in a titer of 1:80 (22 days after starting steroids). Approximately three months after starting steroids, NMDA-R IgG antibodies were no longer detectable in the serum using the fixed-cell-based assay, but the FDG-PET and DMI results showed no relevant improvements (FDG-PET revealed only a minimal decrease in hypermetabolic changes parieto-occipitally; DMI even tended to show increasing frontal edematous changes). The patient was clinically completely asymptomatic at this time. Vitamin D was supplemented to reach high normal levels (> 40 ng/ml).

## Discussion

The patient in this case study presented with a relapse of an acute polymorphic psychotic syndrome of a suspected autoimmune origin.

From a diagnostic perspective, the patient did not fulfill the international consensus criteria for a probable/definite diagnosis of NMDA-R encephalitis during both episodes (1) or even probable/definite autoimmune psychosis (4), especially as no NMDA-R IgG antibodies were identified in the CSF (1, 2) and the CSF and EEG findings - which are usually also conspicuous in NMDA-R encephalitis - were normal (2). Nevertheless, more than five years ago, when parallel confirmative testing in different assay systems was not established, we initially suspected a mild type of NMDA-R encephalitis and discussed whether the lack of detectability in the CSF using fixed-cell-based assays could be due to low antibody levels in the CSF, which could also be consistent with the “milder” oligosymptomatic clinical manifestation (8). Furthermore, we discussed whether the antibodies, if present in small amounts, could all be bound to brain tissue, which could act as an “immunoprecipitator” (24). In the second episode, the fixed-cell-based assays were again positive in the serum in different laboratories (and negative in the CSF). In the reference laboratory of Prof. Dalmau in Barcelona (using samples from one month after initial symptom onset in the second episode, fixed-cell-based assays in serum were still positive at that time), no NMDA-R antibody reactivity was found in the live-cell-based assay (which is a more sensitive assay; Jézéquel et al., 2017) in the serum and CSF. The tissue-based assay – as a confirmatory test to prove brain binding (2) – was also negative for NMDA-R IgG antibodies. A very sensitive tissue-based assay on unfixed mouse brain slices (11–13) in serum and CSF was also negative. Therefore, in retrospect, we no longer assume NMDA-R encephalitis, since no antibodies were detectable in the CSF using a spectrum of sensitive tests and no brain binding was observed in the tissue-based assays (although the serum NMDA-R antibodies in the fixed-cell-based assay were still positive in parallel, with titers of 1:80 during the time of lumbar puncture). From a pathophysiological perspective, the NMDA-R antibodies must have reached the central nervous system to have exerted direct antibody effects such as receptor cross-linking and internalization and thus reduced synaptic excitatory neurotransmission (1, 2, 25–27). Otherwise, the results should have shown at least corresponding binding patterns in the tissue-based assays. As neither was the case here, NMDA-R IgG antibodies in the fixed-cell-based assay could have been a false-positive result (earlier reported in 2–14% of cases; 2). This case demonstrates the clinical complexity of some psychiatric cases and the importance of a comprehensive diagnostic workup including different assay systems in comparable situations.

Some special features of the case, suspected to have an autoimmune cause, nevertheless exist:

1. Both episodes occurred with acute polymorphic onset after a possible autoimmune trigger, which is an unusual combination for a primary schizophreniform psychosis. Vaccination with the Tdap-IPV booster vaccine could have triggered B cells in the first episode (8), and a severe COVID-19 infection could have triggered the immune system in the second episode (13).
2. A partially self-limiting clinical course with a drop in serum NMDA-R IgG antibody titers (possibly with decreasing immune stimulus) was observed in both episodes (without antipsychotics and even before steroids), although complete remission did not occur by itself (in the first episode, this was documented over approx. 2 months). Therefore, a steroid pulse was administered in both episodes under the initial assumption of an autoimmune cause.
3. The FDG-PET findings showed an encephalitic pattern in the first episode with frontal hypermetabolism compatible with NMDA-R encephalitis with frontotemporal-to-occipital gradient, which was earlier described in patients with NMDA-R encephalitis (28–31). Accordingly, it could also have been a different form of autoimmune encephalitis (e.g., with a novel neuronal antibody). Advanced MRI using DMI showed alterations compatible with neuroinflammation; however, similar DMI changes were also observed relatively globally in the gray matter of post-COVID-19 patients (15), so DMI changes could also have occurred post-infection. In the second episode, the FDG-PET alterations were still pronounced (although less emphasized than in the first episode). This would not match with the study results in post-COVID-19 syndromes, where hypometabolic (not hypermetabolic) signals were rather found frontotemporally (32). As the FDG-PET findings did not clearly change in the follow-up, despite clinical improvement and steroid treatment, they could possibly also correspond to “normal” findings in this patient (the same trend was seen with regard to the DMI findings). Conversely, the findings could be interpreted as residual findings.
4. Research mass spectrometry analyses of CSF samples identified a combination of neurotransmitter-precursor shortages, increased tryptophan turnover into quinolinic acid, and a low glucose and lactate status. These findings suggest a possible immune-triggered, microglia-driven brain process (33, 34). A potential co-existing GLUT1 energy failure (35) or a vitamin B6-dependent tryptophan conversion block (36) might be alternative contributors. The elevations of quinolinic acid observed in this case would be compatible with neuroinflammatory processes (34). Quinolinic acid is a tryptophan metabolite produced by activated microglia and infiltrating macrophages during central nervous system (CNS) inflammation (33, 34). While quinolinic acid has no known inflammatory role outside the CNS, it is neurotoxic within the brain by binding to NMDA receptors (33). Increased levels of quinolinic acid have been detected in inflammatory CNS diseases such as meningitis (33, 34). Thus, a viral cause following a COVID-19 infection cannot be excluded as a possible trigger.

In conclusion, NMDA-R encephalitis was no longer assumed. Instead, an acute polymorphic psychotic syndrome with an alternative form of autoimmune brain involvement was suspected. Therefore, high normal levels of vitamin D due to its relapse-preventing effect on autoimmune diseases (37, 38) were reached using substitution. Furthermore, a psychotherapeutic crisis plan was developed between the patient and a clinical psychologist, and—using an exploratory approach—six-monthly serum NMDA-R antibody monitoring (as a suspected nonspecific autoimmune marker) was initiated.

From a research perspective, this case taught us that a thorough diagnostic workup should be carried out in similar cases so that no false diagnoses of NMDA-R encephalitis are made (as we also did in 2019; 8). In contrast, the findings are compatible with previous studies in psychiatric collectives, which have also found NMDA-R antibodies in serum in patients with schizophreniform psychoses. These cases were even associated with fewer negative symptoms and better functional outcomes, as observed in the present patient (5, 6). However, the unclear imaging findings in the patient also suggest that antibody-positive patients should be subject to a broad diagnostic work-up in the future so that autoimmune brain involvement is not overlooked. By producing monoclonal NMDA-R antibodies in laboratory work, investigations of the exact target antigens, mutations or structural analysis of epitopes could also be carried out in such cases (25, 27, 39). Results may then help to explain why the fixed-cell-based assays became positive in serum and tissue-based assays remained negative. In previous laboratory studies, NMDA-R antibodies from patients with schizophrenia did not compete for binding on the native NMDA-R, but altered the surface dynamics and nanoscale organization of the receptor (40). Therefore, the pathophysiological processes involved in some psychosis cases could be different from those involved in NMDA-R encephalitis.

A limitation is that the patient could have suffered from a primary psychosis (41), as no classical symptoms and findings were evident for NMDA-R encephalitis (1) and the further findings were non-specific. Advanced antibody testing was not performed immediately after the onset of the second episode, but only after approximately one month.

In summary, this follow-up case report shows the importance of comprehensive diagnostic approaches in similar cases. In addition, it demonstrates the need for further laboratory and clinical research in the field of immunopsychiatry.

## Data Availability

The original contributions presented in the study are included in the article, further inquiries can be directed to the corresponding author/s.
